# Innovation in Precision Cardio-Oncology During the Coronavirus Pandemic and Into a Post-pandemic World

**DOI:** 10.3389/fcvm.2020.00145

**Published:** 2020-08-14

**Authors:** Sherry-Ann Brown, June-Wha Rhee, Avirup Guha, Vijay U. Rao

**Affiliations:** ^1^Cardio-Oncology Program, Division of Cardiovascular Medicine, Medical College of Wisconsin, Milwaukee, WI, United States; ^2^Stanford Cardiovascular Institute, Stanford University, Stanford, CA, United States; ^3^Harrington Heart and Vascular Institute, Case Western Reserve University, Cleveland, OH, United States; ^4^Franciscan Health, Indianapolis, Indiana Heart Physicians, Indianapolis, IN, United States

**Keywords:** innovation, precision, cardio-oncology, artificial intelligence, machine learning, big data, digital health, telemedicine

## Introduction

Almost 2 million new cancer diagnoses will be made and more than 600,000 cancer deaths will occur in 2020, the equivalent of 5,000 new cases and 1,600 deaths daily (1). Juxtaposed with these staggering numbers is the prevalence of ~17 million cancer survivors in the United States, with a projected estimate of 26 million in 2040 (2); advances in cancer treatments have significantly improved survival across cancers. With growing numbers of survivors comes a growing number of individuals at risk for or living with higher rates of cardiovascular disease than in the general population. In fact, cardiovascular disease is a leading cause of death in cancer survivors, second only to cancer recurrence or the development of new primary cancers (3). Consequently, Cardio-Oncology has emerged as a new field of medicine to specifically address cardiovascular care of cancer patients and survivors, with a particular focus on prevention.

**Figure 1:**
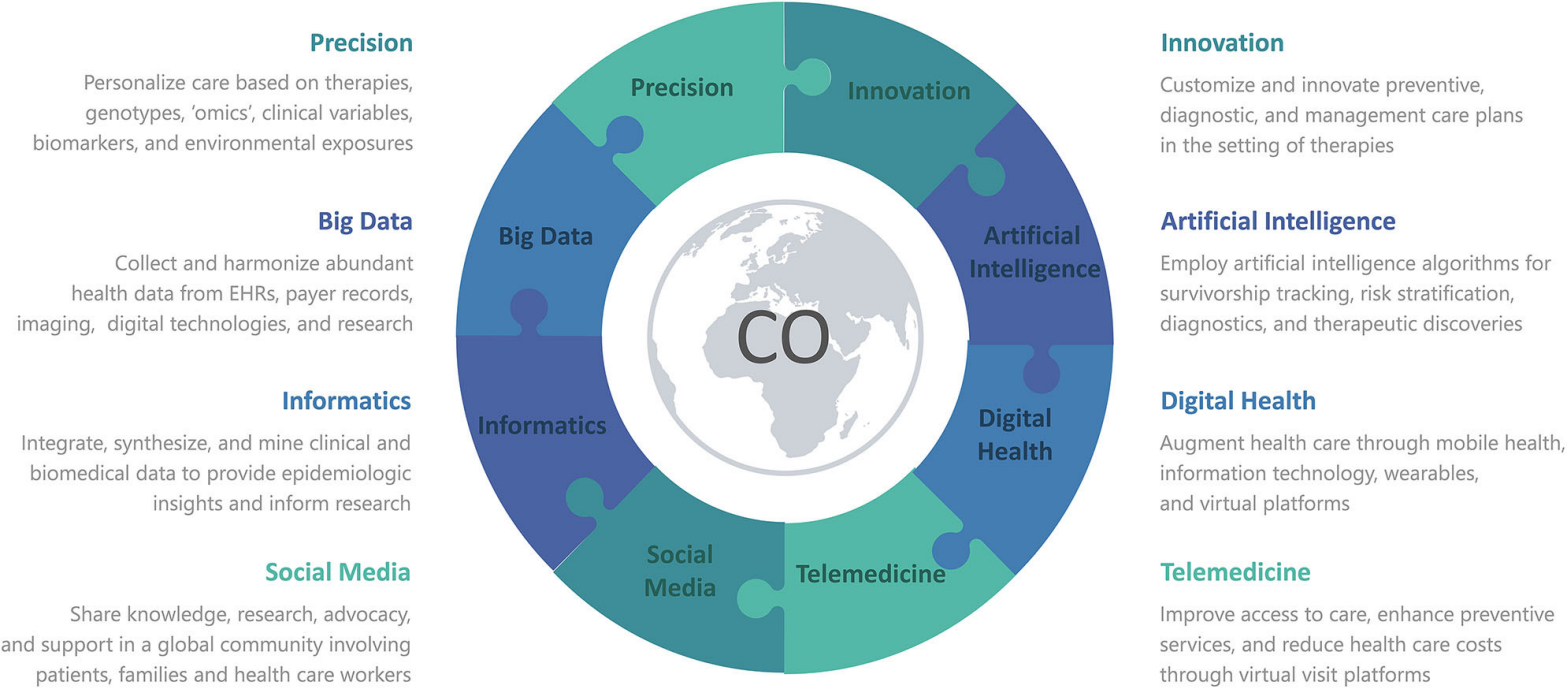
Various forms of Innovation advance Precision Medicine in Cardio-Oncology or COVID-19, with the use of Informatics to integrate Big Data from Artificial Intelligence, Digital Health, Telemedicine, and Social Media. CO denotes either Cardio-Oncology or COVID-19 or both.

Reminiscent of cardiovascular toxicities from cancer therapies, the recent coronavirus disease of 2019 (COVID-19) pandemic is a clear example of how cardiotoxicities can arise unexpectedly and how adaptable clinicians need to be to deal with a constant flow of new cardiotoxic agents and their complications. The severe acute respiratory syndrome coronavirus 2 (SARS-CoV-2) has arisen as an emergent cardiotoxic agent, underlying COVID-19. By July 2020, more than 18 million confirmed cases and 600,000 deaths had been reported globally (4). In positive cases, direct and indirect cardiovascular (CV) injury has been noted as a prominent feature (5, 6), mediated by hypoxia, inflammation, demand ischemia, microvascular dysfunction, or thrombosis (7–10). Around the world, our patients have been physically and socially distancing themselves from others and avoiding physical entrance of health care facilities, in order to limit exposure in COVID-19. Correspondingly, health care institutions have restricted non-emergent in-person visits, to curb the rates of morbidity and mortality from COVID-19. Individuals with known CV disease or risk factors have been at greater risk of morbidity and mortality in COVID-19 (11–16), as is similar in Cardio-Oncology (17). Therefore, there is an urgent need for various avenues of innovation to predict cardiovascular risk and customize preventive, diagnostic, and management care plans in the setting of cancer therapies, especially during the pandemic and beyond. Here, we briefly describe forms of innovation implemented during the pandemic, as well as innovative tools being explored for utility beyond the pandemic (Figure 1).

## Innovation During the Pandemic

### Digital Health

Digital health technologies include mobile health (mHealth), wearable devices, health information technologies, wireless technologies, virtual platforms and applications, telehealth, telemedicine, artificial intelligence, machine learning, and personalized medicine, with a common goal of improving health care outcomes and efficiency (18). With more and more personalized health and lifestyle information available through digital technologies, care providers are better able to monitor patients' conditions in real time or by retrieving remote data recently stored by patients' local devices, identify treatment side effects, and personalize prevention and intervention strategies. Digital technologies can also empower and engage patients to proactively monitor their health while preventing unnecessary hospital visits, which is especially critical in times of a pandemic such as COVID-19 (19). With the implementation of shelter-in-place and subsequent rapid adaptation of virtual visits during this COVID-19 outbreak, the ability to remotely monitor patients' clinical conditions through digital technologies has become more important than ever.

Remote monitoring can enhance our care of cancer patients and survivors. For example, a wearable cardiac rhythm monitoring device such as an Apple watch can detect abnormal heart rates or rhythms (20). As atrial fibrillation is a common side effect of various cancer therapies, including multiple classes of novel tyrosine kinase inhibitors, the ability to detect this rhythm abnormality early and accurately through a wearable cardiac rhythm monitoring device would have an important impact in the ongoing care, as well as future treatment decisions, for cancer patients (21). Abnormal rhythm strips detected from these devices can now be shared and reviewed by the care team, which can potentially alter the treatment course and prevent undesirable toxicities. Another example is virtual cardiac rehabilitation and monitoring (22). Cancer therapies such as doxorubicin can cause myocardial injury and cardiac dysfunction, requiring close monitoring and preferably a tailored rehabilitation program as patients work to recover (23). Virtual rehab programs enable remote collection and evaluation of health data such as activity levels, blood pressures, heart rate/rhythms, and weight, which can be reviewed and acted upon when necessary by health care providers, allowing cancer patients and survivors to safely and efficiently recover from their cardiac complications. This has been of particular importance during COVID-19 pandemic, as many have avoided or limited outdoor physical activities. Guided virtual indoor rehabilitation would allow cancer patients and survivors to continue physical conditioning and rehabilitation and thereby remain physically active during the pandemic.

Digital technologies can provide the unique ability to quickly scale to larger populations with less time, money, and resources, and thereby facilitate near real-time data insights that allow for point-of-service execution (24). These technologies will be critical in caring for cancer patients and survivors, as their numbers continue to increase, with more cancer therapies and related cardiotoxicity profiles dynamically changing daily.

### Telemedicine

Telemedicine or telehealth is the delivery of healthcare at a distance utilizing various technology platforms. Health care systems have recently devoted increased resources to implementation of telemedicine or telehealth services during the pandemic, building upon prior goals of improving access to specialty care, enhancing preventive services, reducing health care costs, and improving patient and provider safety and satisfaction (25, 26). Numerous platforms have been actualized (27), including those embedded within electronic health records (e.g., In-Touch through EPIC) or third-party vendors such as *Doxy.me* or Zoom. Many of the software solutions are cloud-based, accessible (requiring only a desktop, tablet, or smartphone), and free, and have prioritized being HIPAA (Health Insurance Portability and Accountability Act)-compliant. However, security concerns have arisen with some vendors, leading to more careful attention to cybersecurity to enable telemedicine. Indeed, to facilitate wide-spread adoption of telemedicine, great emphasis on protection of patient information through cybersecurity technology will be key, in tandem with the persistence of government-supported regulations and initiatives.

Adoption of these platforms has been expedited during the pandemic to dramatically reduce in-person clinical visits and conform to social distancing (28). The US federal government has taken steps to support rapid and widespread utilization of telemedicine by allowing cross-state accreditation, developing new telemedicine billing codes, and temporarily reducing strict privacy restrictions while still protecting patients and providers (29). As a result, practices across the country converted to virtual clinics in a matter of weeks. This conversion has been especially important for our cardio-oncology patients, who are particularly vulnerable, given their high cardiovascular disease burden and immunocompromised states placing them at high risk for COVID-19 (30). Cardio-oncology, which relies heavily on the patient history and our understanding of cancer therapy regimens, is ideally suited to make the transition to telemedicine.

A recent report described the virtual adaptation of a Cardio-Oncology clinic (31). Suggestions for ensuring a successful patient-centered telemedicine visit include making eye contact with the patient, thanking the patient for inviting the provider into their home, and intentionally offering an excellent “webside” manner. It may become commonplace for initial cardio-oncology consultations to occur via a virtual platform, with follow-up visits (e.g., for reports on home blood pressures) occurring via telephone or secure messaging. Telemedicine could optimize cardio-oncologic care with (i) three-way video or teleconferences enabling the patient/oncologist/cardio-oncologist to collaboratively initiate treatment plans and monitoring algorithms similar to virtual multidisciplinary tumor boards, (ii) follow-up visits to monitor for hypertension and review cardiac function on surveillance imaging in patients on active cancer therapy, and (iii) access points to specialized cardio-oncologist expertise for oncologists in the community (32). While COVID-19 has exposed many limitations in our healthcare system, the expansion and integration of telemedicine in clinical practice will undoubtedly continue to play a larger role than ever before (33), and we are well-poised in cardio-oncology to help lead the way and benefit from this widespread adoption. The Association of American Medical Colleges has submitted a letter to the Centers for Medicare and Medicaid Services to appeal for the permanence of the widescale telemonitoring provisions made during the pandemic^1^. Bipartisan senators and other groups have also submitted similar letters in their respective spheres. With support from the senate and other governmental bodies, telemedicine will likely prevail after the pandemic.

### Social Media

Social media provides an incredible opportunity for healthcare workers and patients and their families to share and exchange knowledge, research, and advocacy, and support in a global community. Spreading education and awareness on social media can propagate messages for prevention and disseminate discoveries and innovation (34–36). Online resources provide timely and timeless sources of information that can have tremendous impact for patients and health professionals if curated appropriately and accurately.

Social distancing during the COVID-19 pandemic has led to enhanced experiences of social networking online, as both patients and healthcare workers reached out to strengthen community and further buttress knowledge, for example, on Facebook (Facebook, Inc.; www.facebook.com) and Twitter (Twitter, Inc.; www.twitter.com) (37–42). Community and sharing of information were developed by patients among each other, healthcare workers among each other, and with cross-pollination between the two sets of communities as healthcare workers themselves became patients in the pandemic.

Social media integrated with the rise of telemedicine or telehealth, with creation of the hashtag #TelemedNow on Twitter (43), with associated twitter chats and threads. Individuals from various public and private healthcare sectors joined in the real-time discussions to share stories, successes, and challenges from implementing telemedicine or telehealth in response to COVID-19.

At no point did the impact of social media wane during the COVID-19 pandemic. In fact, social media became even more important for innovation, information, and prevention. Preventive Cardio-Oncology, Precision Cardio-Oncology, and other Cardio-Oncology tweets would spread across Twitter before the pandemic. These messages continued throughout the time of COVID-19, as preventive and innovative cardio-oncologic care of our patients remained of paramount value. Several pandemic-related Cardio-Oncology papers have been rapidly published, including one on the role of telehealth (31). Within a few hours, this paper was being disseminated on social media, to be assessed and validated or rebutted by healthcare workers and patients alike. Cardio-Oncology can learn much from the time of COVID-19. Rapid and persistent propagation of information can place relevant details in the palms of cancer patients and survivors and their healthcare providers in real-time. Such innovation should help protect the hearts and wellness of our patients and clinicians.

## Innovation Beyond the Pandemic

### Artificial Intelligence

Much of digital health is driven by artificial intelligence. Remote monitoring, wearables, mobile health (mHealth), voice apps, voice analysis, and drones all depend on the simulation of human intelligence. All of these components can be useful in both the COVID-19 pandemic and the practice of Cardio-Oncology. Many of these technologies are also being explored for various scenarios in cardiology (44–51), and have great clinical utility for cardio-oncology and COVID-19. Remote monitoring from wearable biosensors and mHealth is being investigated to improve outcomes in heart rhythm and heart failure and other cardiovascular conditions (44, 46–50), and may have utility for COVID-19 (19, 52–58) and Cardio-Oncology (59–61). Voice apps and voice analysis have shown promise in cardiology for heart failure, ischemic heart disease, pulmonary hypertension, and other forms of cardiovascular disease (45, 62–64), as well as cardio-oncology (65), and have been considered for COVID-19. Drones built on artificial intelligence are being used to deliver healthcare equipment, medicines, personal protective equipment, and food, especially to remote areas with high rates of illness with COVID-19, and are also being dispatched to dense urban locations to urge pedestrians to maintain social distancing (66–68). Similar drones could be used to transport healthcare equipment, medicines, and supplies to cancer patients and survivors with limited mobilities and care support. Particularly in rural America, where advanced cancer and heart care services are limited (69), drones may facilitate delivery of point-of-care equipment and specialty medicines recommended by cardio-oncologists following remote assessment of cancer patients and survivors through virtual care.

Artificial intelligence algorithms could also be used to track cancer survivors and detect any early signs of cardiovascular risk features, saving lives of those who fought and overcame cancer years before. Other relevant AI applications currently being explored include (1) *in silico* screening to develop novel or repurposed therapeutics, (2) patient tracking by location or geography, (3) online voice apps on smartphones, tablets, and smart speakers to promote drug compliance as well as screen for new symptoms or disseminate educational information, and (4) big data predictive analytics to enhance prediction of disease incidence, severity, spread, and recovery (42, 70–80). There is a myriad of lessons to be learned from incredible technological progress being made during these epic times. The algorithms created or adapted for the era of COVID-19 should remain available for use and wide application in medicine, and especially in cardio-oncology, far beyond the pandemic.

Artificial intelligence has also been integrated with social media and interaction during the pandemic (42, 81). Twitter chatter has been monitored to assess individuals' self-reports of COVID-19 symptoms, testing experience, and recovery from illness (81). Gaps in care for symptomatic individuals have been revealed, due to limited testing capacity, and this has likely compromised accurate case counts of COVID-19 positivity at the city, state, and national, and global level. Interactive chatbots have utilized artificial intelligence to spread COVID-19 awareness and education and provide information and patient guidance (42). Analysis of social media chatter could help identify cancer patients and survivors with symptoms suggestive of cardiovascular toxicity and connect them with healthcare resources in cardio-oncology. Monitoring of social media channels could also help recruit patients into cohort studies and build national and international networks to optimize connectivity and care of cancer survivors.

### Precision

Recent advances in multi-omics technologies may help us to collect in-depth large-scale data to better understand disease mechanisms, identify populations at risk, and discover preventive or therapeutic interventions (82). For example, the current state of the sequencing technologies renders whole genome sequencing to be performed in an accelerated and cost-effective (<$1,000) fashion (83). The consequent exponentially increasing genetic knowledge combined with deep cardiovascular phenotyping of cancer patients may allow us to identify genetic variants predicting either increased susceptibility or tolerance for specific drug-induced cardiotoxicity and thereby to risk stratify patients based on their genetic backgrounds (84). The same type of genomic data may also be applied and utilized to identify those at risk for COVID-19 complications. For example, a genome wide association study was recently completed on two case–control panels (835 patients and 1,255 control participants from Italy, and 775 patients and 950 control participants from Spain). The study identified COVID-19 susceptibility genetic loci (3p21.31 gene cluster) which could help risk stratify patients (85).

Additionally, novel biomarker discoveries may be possible through transcriptomics, metabolomics, or proteomics of patients' biological samples (e.g., serum), to complement current imaging-based screening strategies for early detection of cardiotoxicities (86) in cancer and in COVID-19. This is particularly relevant in the era of the COVID-19 pandemic, as we work to avoid clinical encounters or diagnostic studies such as echocardiography that would require in-person interactions (87). More refined biomarkers discovered through multi-omics investigations may allow physicians to closely and accurately monitor cardiotoxicities while minimizing in-person evaluations.

Finally, deeper understanding of ethnic disparities and socioeconomic factors may be achieved through population data-based epigenomics, environmentomics, or populomics, which in turn allows clinicians to assess patients holistically and tailor treatment strategies accordingly (88). Taken together, with accumulating comprehensive omics data, physicians may be able to deliver patients' individualized care based on their cancer therapies, genotypes, phenotypes, biomarker profiles, lifestyle, and surrounding environment, enabling precision cardio-oncology.

### Big Data and Informatics

All aforementioned technologies have the potential to create an ever-increasing volume of data on our patients in the COVID-19 and post-pandemic world. Biomedical and clinical informatics can be useful for combining or mining the data and integrating data sources with the electronic health records. In addition, due to social distancing and reduced in-person work hours, traditional pathways of clinical research have been put on hold or disrupted completely. Big data generated from various government and non-government sources can supplement and help restart some of these endeavors amenable to informatics.

Claims-based information from Medicare registries, as well as Surveillance, Epidemiology, and End Results (SEER) databases, in addition to Truven and Healthcare Cost and Utilization Project (HCUP) datasets, can also reveal epidemiological insights regarding incidence, prevalence, trends, costs, and “codable” outcomes (89–91). The International Classification of Disease (ICD) version 10 and Healthcare Common Procedure Coding System (HCPCS) codes that have been created for COVID-19 will be helpful for capturing large-scale data on signs, symptoms, exposure, testing, diagnosis and treatment of this condition (92, 93). These codes may be used across the globe, including in countries which have nationalized healthcare system repositories like Sweden (94), Denmark (95), and the United Kingdom (96). These repositories can also overcome challenges faced when mining anti-cancer therapy information, since drug coverage in the US is heterogenous among insurance companies, resulting in more variability of administration of particular neoplastic drugs.

Several barriers to meaningful collection and use of big data are being quickly overcome during the pandemic, with rapid data-sharing. Challenges with physical recruitment of study participants for prospective studies have halted some pre-existing clinical trials or cohort studies. However, new trials and paradigms have emerged during the pandemic particularly in cancer patients, to facilitate digital clinical trials and cohort studies based on remote monitoring and virtual care (97, 98). Such paradigms enable novel methodology and also allow for continuation of biomedical inquiry in the midst of COVID-19. These tools will not be limited to the pandemic and will likely enrich our conduct of prospective studies in Cardio-Oncology.

**Figure 2:**
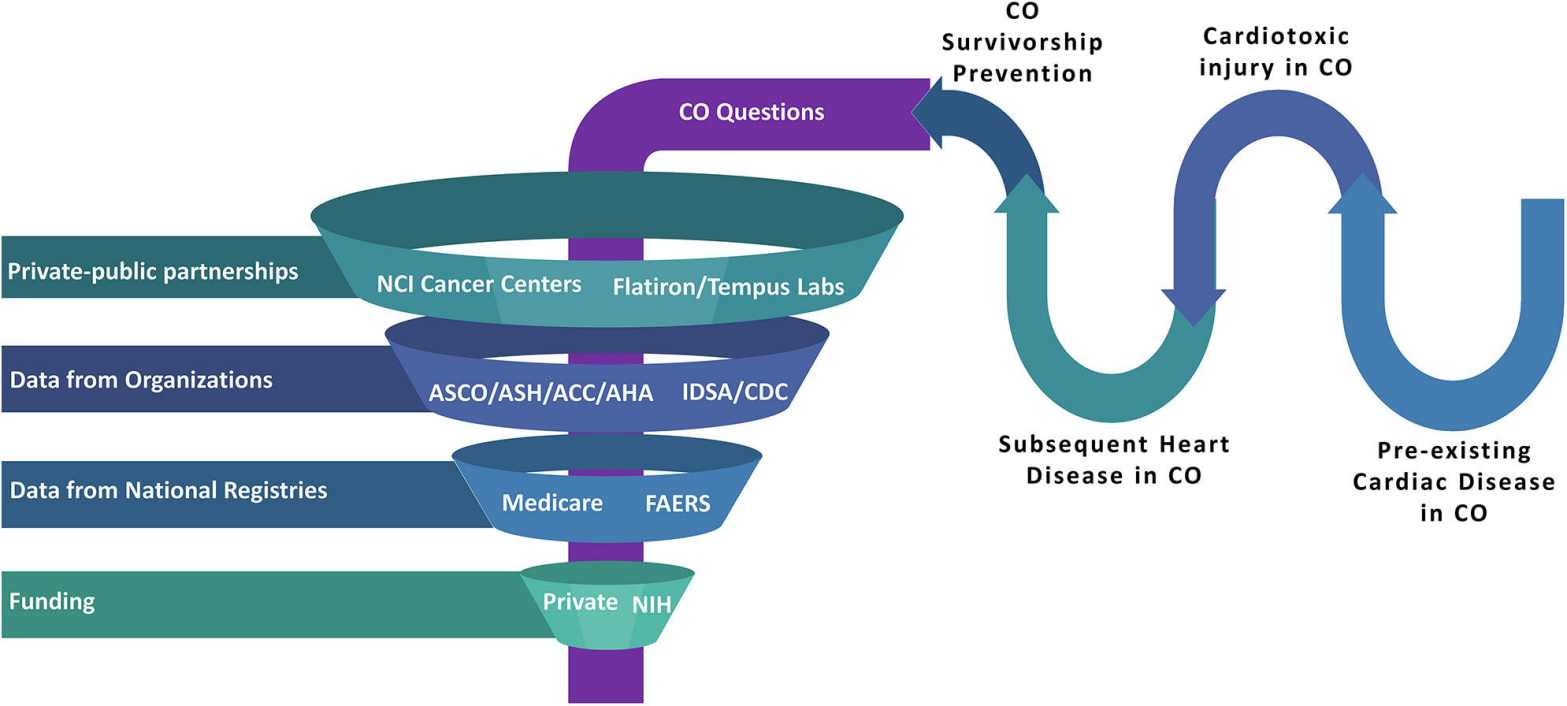
Realms of Big Data such as national medical societies, data science companies (which specialize in patient electronic medical record data management), pharmaceutical industry partners, national databases, and multiple institutions can intersect with CO patient and survivor needs to optimize clinical care and research. This approach can be used to investigate cardiovascular toxic disease or therapy, heart disease predating or as a consequence of CO, survivorship and prevention initiatives, and other relevant themes. Regulatory processes will be needed to ensure preservation of privacy, fairness, inclusiveness, transparency, accountability, and appropriate oversight (79), to ensure the safety of our patients and their protected information. ACC, American College of Cardiology; AHA, American Heart Association; ASCO, American Society of Clinical Oncology; ASH, American Society of Hematology; CDC, Centers for Disease Control and Prevention; CO, either Cardio-Oncology or COVID-19 or both; FAERS, Food and Drug Administration Adverse Event Reporting System; IDSA, Infectious Diseased Society of America; NIH, National Institute of Health. CO denotes either Cardio-Oncology or COVID-19 or both.

Structured multi-pronged approaches should continue to be developed (Figure 2), similar to a vision for integrative and collaborative cardio-oncology practice and research laid out in the 2019 Global Cardio-Oncology summit meeting (99). Collective research and clinical practice targets in precision cardio-oncology could be divided among institutions and societies like the American College of Cardiology or American Heart Association, in partnership with large cancer centers. Industry partners should continue to sponsor clinical trials of anti-cancer therapies. Large oncological organizations such as the American Society of Clinical Oncology or the American Society of Hematology should also participate, and privately owned data science companies [e.g., Flatiron Health Inc. and Tempus Labs Inc. (100)] should create databases which are granular to the study of cardio-oncology epidemiology, multi-omics, and biomarkers to inform basic, translational and clinical research to further these aims. These companies work in the field of data management of patient electronic medical record data into analyzable back ends with heavy focus in the field of oncology. However, in light of the recent major retractions of COVID-19 articles that used a large dataset from a private enterprise, a detailed public reporting of data source architecture, data dictionary, and signed attestation by all authors should be mandated while collaborating with private enterprises.

## Conclusion

The COVID-19 pandemic has dramatically transformed health care and delivery, accelerating and actualizing a wide spectrum of technology solutions. Over the course of just a few weeks, outpatient practices across the country have been converted to virtual clinics to conform to social distancing. Digital technologies have also been rapidly incorporated into clinical care to further complement virtual care. Social media has played more important roles than ever in sharing and disseminating important health care information particularly relevant to cardiovascular complications of COVID-19. Healthcare and biomedical data, as well as precision health, have been assimilated through innovative ways to advance the care of our patients. These advances, along with the lessons learned through our experiences with COVID-19 will undoubtedly reshape our long-term care of patients and survivors in cardio-oncology.

Clinical implementation of these forms of innovation was heralded with the incorporation of “remote patient monitoring” or telemonitoring in the 2016 European Society of Cardiology guidelines for management of heart failure (101). The ACC and AHA have now followed suit and expanded indications for telehealth, remote monitoring, wearables, and other tools in digital medicine throughout the specialty of Cardiology during and after the pandemic (57). New guidelines and recommendations in subsequent years should also encourage the integration of remote monitoring, telemedicine, precision medicine, informatics, and other forms of digital health in electronic health records in Cardio-Oncology, among other medical and surgical specialties. Social media and artificial intelligence should also coalesce with these tools for synergistic monitoring, assessment, and health education. Such integration will help propel optimal care of patients and survivors further along the innovation spectrum in the Digital Post-Pandemic Era.

## Author Contributions

S-AB conceptualized the manuscript. S-AB, J-WR, AG, and VR drafted, edited, and approved the manuscript for publication. All authors contributed to the article and approved the submitted version.

## Conflict of Interest

The authors declare that the research was conducted in the absence of any commercial or financial relationships that could be construed as a potential conflict of interest.

## Footnotes

^1^https://www.aamc.org/system/files/2020-05/ocomm-hca-aamclettertoCMS5132020.pdf
